# Urinary concentrations of neonicotinoid insecticides were related to renal tubular dysfunction and neuropsychological complaints in Dry-zone of Sri Lanka

**DOI:** 10.1038/s41598-021-01732-2

**Published:** 2021-11-18

**Authors:** Kumiko Taira, Tomonori Kawakami, Sujithra Kaushaliya Weragoda, H. M. Ayala S. Herath, Yoshinori Ikenaka, Kazutoshi Fujioka, Madhubhani Hemachandra, Nirmalie Pallewatta, Yoshiko Aoyama, Mayumi Ishizuka, Jean-Marc Bonmatin, Makiko Komori

**Affiliations:** 1 Tokyo Women’s Medical University Medical Center East, Nishiogu, Arakawa-ku, Tokyo, Japan; 2 Toyama Prefectural University, Imizu, Japan; 3 National Water Supply and Drainage Board, Jaffna, Sri Lanka; 4 Hokkaido University, Sapporo, Japan; 5 Northwest University, Potchefstroom, South Africa; 6 Albany College of Pharmacy and Health Sciences, Albany, USA; 7 Arizona State University, Tempe, USA; 8 University of Colombo, Colombo, Sri Lanka; 9 Aoyama Allergy Clinic, Gunma, Japan; 10 Centre National de La Recherche Scientifique, Orléans, France

**Keywords:** Chronic kidney disease, Diagnostic markers, Urinalysis, Pharmacokinetics, Interstitial nephritis

## Abstract

Neonicotinoids are systemic insecticides used since the 1990’s , that possess renal tubular toxicity. We conducted a field-based descriptive study in the North Central Dry-zone of Sri Lanka, where chronic kidney disease (CKD) of unknown etiology has been increasing since the 1990’s. To elucidate the relationship between renal tubular dysfunctions and urinary neonicotinoids concentrations, we collected spot urine samples from15 CKD patients, 15 family members, and 62 neighbors in 2015, analyzed two renal tubular biomarkers, Cystatin-C and L-FABP, quantified seven neonicotinoids and a metabolite *N*-desmethyl-acetamiprid by LC–MS/MS; and we investigated their symptoms using a questionnaire. Cystatin-C and L-FABP had a positive correlation (*p* < 0.001). *N*-Desmethyl-acetamiprid was detected in 92.4% of the urine samples, followed by dinotefuran (17.4%), thiamethoxam (17.4%), clothianidin (9.8%), thiacloprid and imidacloprid. Dinotefuran and thiacloprid have never been registered in Sri Lanka. In High Cystatin-C group (> 70 μg/gCre, n = 7), higher urinary concentration of dinotefuran (*p* = 0.009), and in Zero Cystatin-C group (< LOQ, n = 7), higher *N*-desmethyl-acetamiprid (*p* = 0.013), dinotefuran (*p* = 0.049), and thiacloprid (*p* = 0.035), and more complaints of chest pains, stomachache, skin eruption and diarrhea (*p* < 0.05) were found than in Normal Cystatin-C group (n = 78). Urinary neonicotinoids may be one of the potential risk factors for renal tubular dysfunction in this area.

## Introduction

Chronic kidney disease (CKD) is a global health issue^1^. The causes of CKD, such as diabetes mellitus, hypertension, chronic nephritis, acute kidney injury and nephrotoxins, caused by arsenic and fluoride, have been discussed. However, in several areas of the world, there is a growing concern about CKD of unknown/uncertain etiology (CKDu), which cannot be attributed to those causes^2,3^. For example, in the Dry-zone of Sri Lanka, the epidemic of CKDu has been one of the most serious concerns for public health in the past two decades^4,5^. The clinical features of CKDu in Sri Lanka are shown in Table 1^6,7^.

**Table 1:** Clinical features of CKDu in Sri Lanka.

1. The ratio of male and female is 2.4–3:1, typically male farmers, 40–60 years old, engaged in rice production for more than 10 years in the same area, and are usually poor, but also observed among women and young children
2. The distribution of patients is patchy^a.^. In the urban area with a clean water supply, the prevalence of CKDu is low and in the agricultural areas with ground water use, it is high
3. Hypertension and edema are only seen in the advance stages
4. Urine is hypotonic with β2-microgloburin; and renal pathological change is mainly in tubules and interstitial tissues

^a^For example, in a village, 2–3% of population no less than 18 years old are affected by CKDu, but in the neighboring village only a few kilometers away, no patients are found.

Diagnostic methods in the early stage of the CKDu is still controversial^8^. To confirm the CKDu, a renal biopsy is the gold standard; however, recent studies reveal that the ability of renal tubule reabsorption begins to decrease for at least 10 years preceding the CKDu diagnosis^9^. As urinary biomarkers of renal tubule condition, albumin and low molecular proteins, which is constantly secreted from glomerulus and absorbed by normal renal tubules have been used, e.g. urine albumin to creatinine ratio (UACR)^10–15^. Urinary Cystatin-C and L-type fatty acid–binding protein (L-FABP) are new renal tubular biomarkers draw attention of researchers by their unique dynamics (Table 2)^13,16–19^.

**Table 2:** Comparison of the characteristics of three urinary biomarkers.

Biomarker	Creatinine	Cystatin-C	L-FABP
Molecular weight	113.1 g/mole	13 kDa, protein	14–15 kDa, protein
Origin, physiological	Skeletal muscle	Cell	Liver
Origin, pathological			Proximal tubules under ischemia and oxidative stress
Secretion in nephron	Glomerulus	Glomerulus	Glomerulus, pathological proximal tubules
Reabsorption in nephron	No	Proximal tubule	Normal proximal tubules
Reference value	Not determined	≦70 μg/g Cre	≦8.4 μg/g Cre

As for the etiology, more than 30 factors including pesticides have been discussed (Supplementary Table S1 online). Pesticides and fertilizers are major environmental chemicals that farmers are occupationally exposed to^20–27^. Neonicotinoids are systemic insecticides with competitive modulator actions on nicotinic acetylcholine receptors in invertebrates and vertebrates including mammals^28–30^, while they exert a serious impact on ecosystems in many countries because of their much longer half lives in plants and in the environment (soil and water) compared to those of organophosphate insecticides^31,32^. Acute and chronic neonicotinoid exposure after absorption via the intestines and lungs may cause renal dysfunction as well as systemic symptoms as shown in Table 3^33,34^. Neonicotinoids and the metabolites are detected in human urine samples from healthy volunteers as well as the patients with neonicotinoid intoxication^35–40^. *N*-Desmethyl acetamiprid (DMAP), the phase-I metabolite of acetamiprid, is one of the most frequently detected metabolites of neonicotinoids. To evaluate neonicotinoids in the urine, concentration by volume and creatinine adjusted concentration have been used^35–40^.

**Table 3:** Typical symptoms of neonicotinoid intoxication.

	Acute exposure	Subacute and chronic exposure
Cardiovascular	Tachycardia or bradycardia; hypertension or hypotension	Chest pains; palpitation; electrocardiographic abnormalities
CNS	Low GCS or unconsciousness; sleepiness; dizziness; convulsion; excitation	Headache; finger tremor, recent memory loss; dizziness upon standing; sleeplessness; agitation; fear; anger; abnormal behavior; altered consciousness; dreamy state; sudden change of senses of smell; auditory or visual hallucinations,
Respiratory	Dyspnea or tachypnea; cough; cyanosis; respiratory arrest	Cough
Gastrointestinal	Nausea; vomiting; stomachache; oral-esophageal-gastric erosion	Stomachache; appetite loss; constipation or diarrhea,
Secretion	Diaphoresis or anhidrosis; excessive discharge of saliva and bronchial secretion/mouth dryness	
Pupil	Mydriasis or miosis; abnormal light reflex	Abnormal light reflex
Body temperature	Fever or low body temperature	Fever (> 37°c)
Skeletal muscle	Muscle weakness or spasm; high creatine kinase	General fatigue; shoulder stiffness; muscle pain or spasm or weakness
Others	Metabolic acidosis; leukocytoclastic vasculitis; renal and hepatic disfunction	Oliguria; edema; low or high urine volume; increase of urinary cystatin-c; skin eruptions or itching; reduced or increased body weight

To assess neonicotinoids exposure related to the occupational use, urine sampling at the season for rice cropping is preferable. It is known that the half-lives after acute exposure are no more than 2 days^36,41^, while urinary excretion of DMAP and imidacloprid is slower and more persistent than other neonicotinoids^36,42^. Traditionally, there are two seasons for rice cropping in Sri Lanka, Yala (from April to August) and Maha (from September to January)^43,44^.

The objective of this study is to evaluate the renal tubular function by urinary biomarkers, the actual neonicotinoids exposure by urine, and neonicotinoid related symptoms in the Sri Lanka Dry-zone, and to assess the relationship between them. It can be the first step of appropriate regulation to reduce the pesticide exposure that may cause CKD and other pathology in people living in the CKDu-epidemic area.

## Methods

This study was conducted after the ethic committee’s approval by Tokyo Women’s Medical University (No. 2810R2), as a part of the Sri Lanka CKDu-affected area survey. by Toyama Prefectural University. All methods were carried out in accordance with relevant guidelines and regulations. After obtaining written informed consent from the participants, in May 2015, approx. 50 ml of spot urine samples were collected from 33 residents in Wilgamuwa and Anuradhapura, and in December 2015, 59 residents in Anuradhapura, including CKD patients, and the families lived in the CKDu affected area (local prevalence was more than 10%), and others. CKD was diagnosed at local hospitals, where medical care was accessible, by a decrease of eGFR (less than 60 mL/min/1.73m^2^). No CKD patients had experienced renal biopsy to confirm CKDu until the urine sampling date. The geographic data of sampling area are shown in Table 4. Wilgamuwa and Anuradhapura city include CKDu-affected areas (prevalence of CKDu are more than 10%)^45^. Unpublished database by Water Supply Scheme in Sri Lanka indicated 10,288 CKDu patients (1.2%) was identified, and the prevalence varied from 0 to 16.5% in 692 areas in Anuradhapura in 2013.

**Table 4:** The geographic data of the sampling area in this study.

Sampling area (Divisional Secretariat)	Wilgamuwa	Anuradhapura city
Province	Central	North Central
District	Matale	Anuradhapura
Population of the district	484,531 in 2012	854,602 in 2013
The percentage of households reporting at least one member diagnosed with CKD who resided in the household between 2009 and 2018 in the district	16.7%	18.9%

Systemic questions were administered to each participant by a trained staff about the physical and psychological conditions listed in Table 3 as subacute and chronic symptoms, that were also performed and recorded in the documents. When participants were minors or children, written informed consent was obtained from the next of the kin, caretakers, or guardians on behalf of them. Additionally, we interviewed two clerks in the pesticide sales shops (A and B), who sold pesticides to farmers in Anuradapura to know the kinds of pesticides that were sold in the area.

### Urine analysis

Each urine sample was divided into four plastic tubes, one was analyzed on the day of sampling by trained staff, and other three samples were kept in a refrigerator. Out of these samples one was sent to Hokkaido University (Sapporo, Hokkaido, Japan) and kept in a freezer at − 20 °C for liquid chromatography-tandem mass spectrometry (LC–MS/MS) analysis. Another one out of the three samples were sent to a commercial laboratory IKAGAKU (Kyoto, Japan) to quantify urinary Cystatin-C and creatinine. The last one was used to analyze L-FABP and trace minerals, and the method and the result was reported in the previous publication^46^.

### Simple urine chemistry analysis on the day of sampling

Glucose, protein, bilirubin, urobilinogen, pH, blood, ketone, nitrate, leukocyte, albumin was analyzed with dipstick (Uropiece ® Toyo Roshi Kaisha, Ltd.) and recorded. The specific gravity of each sample was also recorded in the May 2015 survey.

### Quantitative analysis of neonicotinoids and a metabolite by LC-ESI/MS/MS

## Materials

Acetamiprid, dinotefuran, imidacloprid, nitenpyram and thiacloprid were purchased from Kanto Chemical Corp. (Tokyo, Japan). Thiamethoxam was purchased from Dr. Ehrenstorfer (Augsburg, Germany). Clothianidin, clothianidin-d3, dinotefuran-d3, imidacloprid-d4, thiacloprid-d4, thiamethoxam-d4, and *N*-desmethyl-acetamiprid (DMAP) were purchased from Sigma-Aldrich (St. Louis, MO, USA). Acetamiprid-d6 and nitenpyram-d3 were purchased from Hayashi Pure Chemical Ind. (Osaka, Japan). Acetonitrile, dichloromethane formic acid, ammonium acetate and distilled water were all HPLC grade and were purchased from Kanto Chemical (Tokyo, Japan).

### Urine sample preparation

The urine sample preparation was performed according to Ichikawa et al.^47^. A liquid chromatography-electrospray ionization tandem mass spectrometry (LC-ESI/MS/MS) system (Agilent 6495B, Agilent Technologies, Santa Clara, CA, USA) equipped with a Kinetex Biphenyl column (2.1 mm ID × 100 mm, ϕ2.6 μm, Phenomenex, Torrance, CA, USA) was used for quantitative analysis. For mass spectrometry, multiple reaction monitoring (MRM) was programmed. The MRM transition of precursor and product ions are shown in Table 5. The recovery efficient of each neonicotinoid and its metabolites ranged from 80 to 120%. The reproducibility of the analysis system was confirmed in the duplicate analyses of each sample, with a relative standard deviation (RSD) of 10% for all the compounds.

**Table 5:** The MRM transits, retention times, recovery % and LOQ of seven neonicotinoids and DMAP.

Name	MRM (m/z)	RT (min)	Recovery rate (%)	LOQ (μg/L)
Imidacloprid	256.00 > 209.05	17.3	87.0 ± 2.7	0.5
Acetamiprid	223.00 > 126.00	16.2	80.2 ± 2.9	0.05
Nitenpyram	271.00 > 126.05	8.9	88.6 ± 4.6	0.5
Thiacloprid	252.90 > 126.05	19.1	92.9 ± 1.8	0.05
Thiamethoxam	291.90 > 211.00	14.0	116.7 ± 7.9	0.125
Clothianidin	249.90 > 132.05	16.1	91.8 ± 3.7	0.125
Dinotefuran	203.00 > 129.10	8.2	92.6 ± 2.8	0.125
DMAP	208.90 > 126.05	15.2	87.6 ± 5.4	0.05

*MRM* multiple reaction monitoring; *RT* retention time; *LOQ* limit of quantification; *DMAP N*-desmethyl acetamiprid.

### Quantification of neonicotinoids and a metabolite, DMAP

Seven neonicotinoids and DMAP were analyzed in each sample. Six deuterium-labeled neonicotinoids were used as internal standards. Quantification of the neonicotinoids and DMAP was carried out by the internal standard method. Five calibration points were set at 0.5, 1.25, 2.5, 3.75 and 5 ppb, whereas the internal standard was used to 5 ppb at all calibration points.

### Quality control and quality assurance

Quality control and quality assurance were performed according to Ichikawa et al.^47^. A mixture of six deuterium-labeled neonicotinoids was spiked into samples as an internal standard prior to sample preparation and extraction. Quantification was performed using five calibration points and the average coefficients of determination (*r*^*2*^) for the calibration curves were ≥ 0.995. The analytical method was checked for precision and accuracy. Limits of quantification (LOQs) were calculated based on 3*SD*/*S* (*SD* is the standard deviation of the response of seven replicate standard solution measurements and *S* is the slope of the calibration curve). Recovery % and LOQs (μg/L) of the analytes are given in Table 5.

### Statistical analysis

All statistical analyses were performed in StatPlus version 7.3.32 (AnalystSoft Inc. 2020). To calculate geometric mean of each neonicotinoid and a metabolite concentration, less than LOQ was assumed the half of LOQ. Comparisons of categorical data between two groups were performed by Chi-square test and comparisons of numerical data between two groups by t-test. For comparison of the groups with sample number less than 8, a nonparametric method, Mann–Whitney U test was also applied. The ***p*** value threshold for statistical significance was set at 0.05.

## Results

### The demographic data of the volunteers and basic urinary findings

Data are shown in Table 6. Most of the CKD patients were male, 75%, (in healthy participants 35.1%, *p* < 0.001, Chi-square test) and the age was older, 54.9 ± 13.1 years old (non-CKD participants 40.5 ± 17.7 years old (mean ± SE), *p* = 0.009, t-test).

**Table 6:** Demographic data of 92 participants.

	May 2015			Dec 2015			
	CKDs	Families	Neighbors	CKDs	Families	Neighbors farmers	Neighbors not farmers
N	9	5	19	6	10	12	31
Male	7	2	10	4	2	5	8
Age (mean ± SD)	50.0 ± 10.0	25.6 ± 15.5	33.4 ± 16.2	58.5 ± 14.0	51.0 ± 11.5	52.3 ± 9.0	38.9 ± 19.2
**Area of residence**							
Wilgamuwa	5	1	2	0	0	0	0
Anuradhapura	4	4	17	6	10	12	31
**Occupation**							
Active farmer	No data	No data	No data	0	5	12	0
Retired farmer	No data	No data	No data	5	0	0	0
Not farmer	No data	No data	No data	1	5	0	31

CKDs: CKD patients; Families: CKD patients’ family members; Neighbors: healthy individuals living in Wilgamuwa and Anuradhapura.

No remarkable difference between 15 CKD patients and 77 healthy participants was observed in urinary blood detection (40%, 52.9%, *p* = 0.41, Chi-square test); acidic dominant pH (5.7 ± 0.3, 5.7 ± 0.6, *p* = 0.51, Chi-square test); low urinary creatinine concentration no more than 0.5 g/L (33.3%, 29.9%, *p* = 0.92, Chi-square test); low gravity less than 1.005 (55.6%, 20.8%, *p* = 0.31, only performed in May); and UACR, creatinine adjusted albumin no less than 30 mg/g Cre (33.3%, 3.6%, *p* = 0.06, Chi-square test). Urinary glucose was not detected in CKD patients. The details of the urine analysis result are shown in Supplementary Table S2 online.

### Renal tubular biomarkers

**Figure 1:**
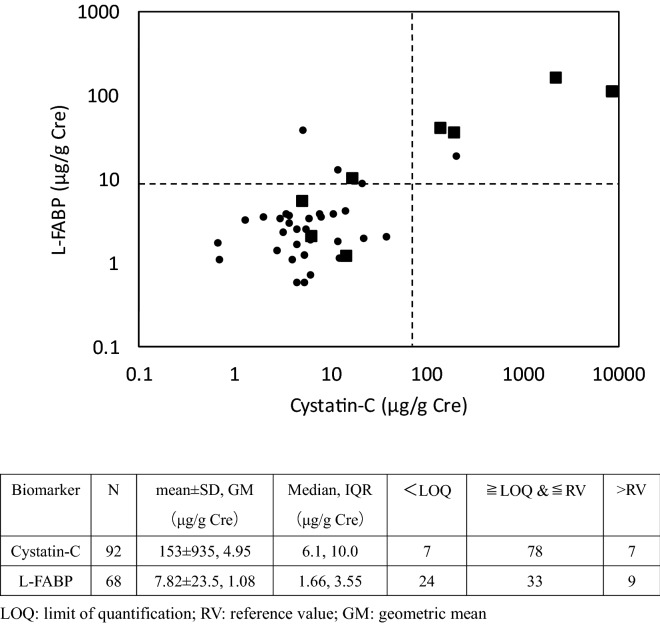
Relationship between L-FABP and Cystatin-C of participants. Black squares stand for CKD patients, small black circles stand for other participants. Broken lines indicate the reference value of markers, L-FABP 8.4 μg/g Cre and Cystatin-C 70 μg/g Cre. Log_10_(L-FABP) = 0.536 × Log_10_(Cystatin-C) + 0.0193, r = 0.75, *p* < 0.001.

The statistical data are shown in footnote table in Fig. 1. L-FABP analysis was in only on 68 samples, because the sample volume was limited. The linear correlation between Cystatin-C and L-FABP are shown in Fig. 1 (r = 0.75, *p* < 0.001, Spearman’s rank correlation test, n = 40). Interestingly, in seven cases of non-CKD participants, Cystatin-C concentrations were less than the limit of quantification. We divided all participants into three groups by Cystatin-C value; 1. Zero CysC group: the participants with Cystatin-C equal to or less than the LOQ (n = 7), 2. Normal CysC group: the participants with Cystatin-C more than the LOQ and no more than reference value (n = 78), and 3. High CysC group: the participants with Cystatin-C more than the reference value (n = 7). Their toxicological profiles are shown in Table 7. We found the urinary creatinine concentration was significantly lower in the Zero CycC group than in the normal CysC group, but not L-FABP.

**Table 7:** Demographic data of three groups classified by Urinary Cystatin-C concentration.

Group	Zero CysC (A)	Normal CysC (B)	High CysC (C)	*p* value^a^ A vs B	*p* value^a^ B vs C
Cystatin-C (μg/g Cre)	< LOQ^b^	LOQ-70	> 70		
N	7	78	7		
Male/female	1/6	32/46	6/1	0.16	**0.022**
CKDs	0	10 (7/3)	5 (4/1)	0.31	**0.0001**
Families	1 (0/1)	14 (4/10)	0		
Neighbors	6 (1/5)	54 (21/33)	2 (2/0)		
Age (mean ± SD)	36.3 ± 15.3	41.9 ± 17.6	61.1 ± 10.2	0.45	**0.004**
UACR > 30	0%	1.3%	28.6%	0.76	** < 0.001**
Urine blood positive	71.4%	43.6%	57.1%	0.17	0.49
Urine pH (mean ± SD)	5.57 ± 0.67	5.72 ± 0.55	5.80 ± 0.45	0.50	0.76
Urine Creatinine					
(mean ± SD, mg/dL)	18.7 ± 8.1	105.2 ± 68.4	74.4 ± 44.9	**0.0013**	0.25
Urine L-FABP					
(mean ± SD, μg/g Cre)	4.72 ± 4.91	1.99 ± 3.08	65.9 ± 54.1	0.056	** < 0.0001**
Low Urine volume (self-reported)	14.3%	25.6%	28.6%	0.50	0.87
High urine volume (self-reported)	28.6%	41.0%	57.1%	0.52	0.41
**Estimated pathology**					
Glomerular damage	No	No	Yes		
Proximal tubule reabsorption	Conserved	Conserved	Impaired		
Proximal tubule damage	Likely	Not likely	Highly damaged		
Distal tubule reabsorption	Impaired	Conserved	Impaired		
**Differential diagnosis**					
CKDu	Not likely	Not likely	Likely		
Other CKD	Not likely	Not likely	Possibly		
Tubulointerstitial nephritis	Likely	Not likely	Possibly		

^a^Chi-square test for categorical data, and t-test for numerical data; ^b^LOQ of Cystatin-C was 0.01 μg/L.

### The status of pesticides applied onto the rice paddies

An interview with a clerk in shop A in Mihintale, Anuradapura revealed that glyphosate was commonly used from April to May, MCPA, 3–4 DPA (propanil) and Gulliver (azimsulfuron) from June to July, and Avimavar (imidacloprid), Mospilan (acetamiprid) and Marshal (carbosulfan) in August. However, from September to next March no specific pesticides were sold (Supplementary Table S3 online). A clerk at shop B said “In Anuradhapura District, variations in the pesticide sales by month is not recorded. The reason is both vegetable farmers (throughout the year) and rice farmers buy different pesticides. But the most saleable pesticides are herbicides, such as Kiseki (bispyribac-sodium 40 g/L and metamifop 100 g/L), Ceypectco (MCPA), propanil, and Weed Lactor (a.i. is unknown), and organophosphate insecticide profenofos”.

### Urinary neonicotinoids

The overall detection rates were the highest for DMAP 92.4%, followed by dinotefuran and thiamethoxam 17.4%, clothianidin 9.8%, thiacloprid 3.3%, imidacloprid 2.2% (Table 8). Dinotefuran and thiacloprid were not registered in 2015 in Sri Lanka. The distribution of neonicotinoids and the DMAP concentration was a gamma distribution. The details of urinary neonicotinoids and DMAP concentration in May 2015 and in December are shown in Supplementary Table S4 online. Urinary DMAP was more significantly detected in December 2015 than in May 2015 (Detection rate, in May 81.8%, in December 98.3%, *p* = 0.0042, Chi-square test; mean ± SD, in May 0.50 ± 0.53 μg/gCre, in December 2.45 ± 4.34 μg/gCre, *p* = 0.012, t-test), but the difference of the detection rate was not significant for other neonicotinoids. The detection rate of urinary DMAP was significantly lower in the CKD participants than in others (*p* = 0.007, Supplementary Table S5 online) with no detection of clothianidin or thiacloprid. Uncorrected concentration of dinotefuran and imidacloprid were higher in CKD participants than in others (*p* = 0.009 and 0.031, respectively), and no significant difference was observed in CKD families and neighbors.

**Table 8:** Concentration and detection rate more than LOQ of urinary neonicotinoids and DMAP.

Neonicotinoid	> LOQ (%)	Mean ± SD (ug/L)		Selected percentile (uncorrected^a^, Cre-adjusted^b^)				Max
			GM^c^ (ug/L)	25th	50th	75th	95th	
DMAP	92.4	0.88 ± 1.09	0.46	0.23, 0.20	0.52, 0.60	0.99,1.61	2.49, 7.37	6.63, 21.5
Dinotefuran	17.4	0.10 ± 0.28	0.09	< LOQ	< LOQ	< LOQ	0.62, 0.74	1.65, 11.5
Thiamethoxam	17.4	0.19 ± 0.80	0.09	< LOQ	< LOQ	< LOQ	0.83, 1.42	7.02, 7.10
Clothianidin	9.8	0.05 ± 0.15	0.07	< LOQ	< LOQ	< LOQ	0.28, 0.50	0.81, 3.61
Thiacloprid	3.3	0.01 ± 0.05	0.03	< LOQ	< LOQ	< LOQ	< LOQ	0.31, 1.18
Imidacloprid	2.2	0.11 ± 0.58	0.26	< LOQ	< LOQ	< LOQ	< LOQ	5.47, 5.44
Acetamiprid	0	< LOQ	< LOQ	< LOQ	< LOQ	< LOQ	< LOQ	< LOQ
Nitenpyram	0	< LOQ	< LOQ	< LOQ	< LOQ	< LOQ	< LOQ	< LOQ

*GM* geometric mean; ^a^Unit of uncorrected concentration is ug/L; ^b^unit of creatinine-adjusted concentration is μg/g Cre.

There was no significant correlation between urinary Cystatin-C and urinary concentration of neonicotinoids. However, as shown in Table 9, for the High CysC group, the uncorrected and the creatinine corrected concentrations of dinotefuran were significantly higher than that in the Normal CysC group (*p* = 0.009, *p* = 0.003, respectively, t test), but not for other neonicotinoids (Details are shown in Supplementary Table S6 online). In the Zero CysC group, the average creatinine corrected concentrations of DMAP (*p* < 0.001, t-test), dinotefuran (*p* < 0.001, t-test), clothianidin (*p* < 0.001, t-test) and thiacloprid (*p* = 0.0011, t-test) were significantly higher than those in the Normal CysC group, but in two cases no neonicotinoids were found (Supplementary Table S7 online).

**Table 9:** Concentration and detection rate more than LOQ of urinary neonicotinoids and DMAP (μg/g Cre) in each class of urinary creatinine-adjusted Cystatin-C concentration.

	Zero CysC (A)	Normal CysC (B)	High CysC (C)	A vs B *p* value^a^	B vs C *p* value^a^	Correlation Coefficient^b^
*Detection rate > LOQ%*						
DMAP	71.4%	96.2%	85.7%	**0.007**	0.21	
Dinotefuran	28.6%	15.4%	28.6%	0.38	0.37	
Thiamethoxam	42.9%	20.5%	0%	0.17	0.18	
Imidacloprid	0%	2.6%	14.3%	0.67	0.11	
Clothianidin	28.6%	16.7%	0%	0.43	0.24	
Thiacloprid	14.3%	2.6%	0%	0.11	0.67	
*Concentration, uncorrected (mean ± SD, μg/L)*						
DMAP	1.90 ± 2.31	0.81 ± 0.92	0.58 ± 0.68	**0.013**, 0.38	0.53, 0.44	− 0.08, 0.44
Dinotefuran	0.24 ± 0.45	0.07 ± 0.19	0.33 ± 0.63	**0.049**, 0.28	**0.009**, 0.25	0.01, 0.92
Thiamethoxam	0.10 ± 0.13	0.21 ± 0.87	0	0.73, 0.26	IC, 0.25	− 0.04, 0.73
Imidacloprid	0	0.08 ± 0.62	0.01 ± 0.03	IC, 0.67	0.78, 0.12	− 0.014, 0.89
Clothianidin	0.15 ± 0.31	0.04 ± 0.13	0	0.057, 0.33	IC, 0.25	− 0.05, 0.64
Thiacloprid	0.04 ± 0.12	0.01 ± 0.04	0	**0.035**, 0.098	IC, 0.67	− 0.03, 0.79
*Concentration, creatinine corrected (mean ± SD, μg/g Cre)*						
DMAP	9.20 ± 8.70	1.16 ± 1.98	0.98 ± 1.08	** < 0.001,** 0.068	0.82, 0.99	− 0.06, 0.57
Dinotefuran	2.12 ± 4.32	0.07 ± 0.19	0.42 ± 0.85	** < 0.001,** 0.27	**0.003,** 0.24	− 0.02, 0.88
Thiamethoxam	0.61 ± 0.98	0.26 ± 0.92	0	0.34, 0.14	IC, 0.25	− 0.05, 0.65
Imidacloprid	0	0.04 ± 0.24	0.01 ± 0.03	IC, 0.67	0.79, 0.12	− 0.01, 0.89
Clothianidin	0.63 ± 1.35	0.05 ± 0.15	0	** < 0.001**, 0.27	IC. 0.25	− 0.04, 0.73
Thiacloprid	0.17 ± 0.45	0.004 ± 0.031	0	**0.001,** 0.098	IC, 0.67	− 0.02, 0.84

IC: incalculable; ^a^For > LOQ%, Chi-square test, for neonicotinoids concentration, t test, Mann–Whitney U test (2-tailed); ^b^Pearson correlation Coefficient between neonicotinoids/DMAP and Cystatin C in A, B and C, R, *p* value.

### Subjective symptoms

We obtained physical and psychological complaints related to neonicotinoids exposure using questionnaire from 91 participants including 15 CKD patients, and 76 non-CKD participants (15 CKD family members and 61 neighbors). A data from a neighbor in May 2015 was lost and could not be included in the analysis. The subjective symptoms frequently complained of by 91 participants were recent memory loss (67.0%), muscle symptoms (60.4%), chest pains or palpitation (57.1%), general fatigue (52.7%), anger (51.6%), headache (49.5%), restlessness (34.1%), auditory hallucination (33.0%), and dizziness after standing up (31.9%). In the participants complaining of finger tremor (n = 13, 14.3%), urinary dinotefuran concentration was significantly higher (*p* = 0.002, t test) (Supplementary Table S8 online).

Subjective symptoms that CKD patients complained of significantly more than non-CKD participants were as follows: high urine volume (66.7%), appetite loss (60.0%), reduced body weight (53.3%), finger tremor (46.7%), fever (46.7%) (*p* < 0.001); abnormal behavior (13.3%) and constipation (13.3%) (*p* < 0.05). There was no significant difference in the detection rate of the symptoms in CKD patients between in May and in December. The subjective symptoms complained of by 15 CKD family members significantly more than from 61 neighbors were as follows: muscle symptoms (86.7%) and abnormal behavior (6.7%) (*p* < 0.05) (Supplementary Table S10 online).

In members of the High CysC group, no subjective symptom was more complained of than members of the Normal CysC group. In members of the Zero CysC group, chest pains, stomachache, skin eruption, skin itching, and diarrhea were more frequently complained of than members of the Normal CysC group (*p* = 0.002, 0,006, 0.022, 0.049, and 0.002, respectively), but not high urine volume nor reduced body weight (Supplementary Table S10 online).

## Discussion

We found some significant relationships between the clinical category (CKD or not), urinary Cystatin-C level, urinary neonicotinoids and DMAP levels, and subjective symptoms in this study (Table 10). CKD patients in the CKDu-affected area were characterized by high urinary Cystatin-C, low urinary DMAP detection and higher urinary concentration of dinotefuran and imidacloprid, and seven symptoms (finger tremor, fever, high volume urine, appetite loss, reduced body weight, abnormal behavior and constipation).

**Table 10:** Relationship between CKD diagnosis, high urine Cystatin-C, very low urine Cystatin-C, urine neonicotinoids/DMAP and subjective symptoms related to neonicotinoids exposure.

	CKD	High CysC	Zero CysC	urine neonicotinoids	subjective symptoms
CKD diagnosed at hospital		*p* = 0.0001	*p* = 0.31	*p* < 0.01: high dinotefuran, low DMAP *p* < 0.05: high imidacloprid	*p* < 0.001: Finger tremor, Fever; High urine volume, Appetite loss, Reduced body weight, *p* < 0.05: Abnormal behavior, Constipation
High CysC (> 70 μg/gCre)	+ + +			*p* < 0.01: high dinotefuran	none
Zero CysC (< LOQ)	±			*p* < 0.05: high DMAP, high dinotefuran, high thiacloprid	*p* < 0.01: Chest pains, Stomachache, Diarrhea *p* < 0.05: Skin eruption, Skin itching
urine neonicotinoid (uncorrected)	+ +	+	+		*p* < 0.001: imidacloprid vs Diarrhea, Constipation *p* < 0.01: dinotefuran vs Fever, Finger tremor
subjective symptoms	+ + +	-	+ +	+ + +	

+  +  + : *p* < 0.001, +  + : *p* < 0.01, + : *p* < 0.05, ± : *p* < 0.5.

Urinary Cystatin-C would be useful for subclinical kidney disease in the early stage where UACR fail to detect in the CKDu-affected area. Additionally, we observed very low levels of urinary Cystatin-C in some non-CKD participants. We first suspected that the observation were due to sample preservation rather than the abnormality of their tubular function, because the LOQ of Cystatin-C analysis by the commercial laboratory was low enough for Japanese urine samples and we had never experienced the case with less than LOQ detection in Japan^35^; however, low creatinine level and normal or a little high L-FABP in their urine without proteinuria, complaints of skin symptoms and diarrhea, and their younger age than High CysC group suggested the possibility they had tubulointerstitial nephritis^48^. Further investigation is needed.

The urinary neonicotinoids and a metabolite analysis revealed the environmental exposure was common in Wilgamuwa and Anuradhapura in Sri Lanka, although we had obtained no direct evidence of the dose and the timing of the occupational use in the studied area. Among them, DMAP was detected in almost all urine samples of the participants. In 2017, 658 tons of insecticides including three neonicotinoids, 1298 tons of herbicides, and 664 tons of fungicides were imported to Sri Lanka as formulations^49^. The active ingredients of three neonicotinoid insecticides, were imidacloprid (6.4 tons), thiamethoxam (2.2 tons), and acetamiprid (3.9 tons), two organophosphates, profenofos (97 tons), and diazinon, (11 tons), one carbamate, BPMC (12 tons), one phenylpyrazole, fipronil (1.3 tons), and one antibiotic, abamectin (1.3 tons). In May 2015 (Yala season) and in December 2015 (Maha season), rice seed sowing seems to be performed and pesticides applied to rice paddies, as significant crop production was reported from Anuradhapura and Matale districts according to the national record, while recently frequent draughts diminished the farmers time available for rice cropping^43,44,50,51^.

As the route of environmental neonicotinoids exposure, via intake of drinking water, tea, rice, vegetables and fruits can be considered. The dietary source of acetamiprid exposure does not seem to be tea leaves nor drinking water. We collected the tea leaves that the participants in this study daily consumed, and water samples that they were drinking at the same time, because they traditionally drink milk tea with spice and sugar many times every day. Ten tea leaves samples that 10 CKD patients consumed were analyzed and no neonicotinoids were detected from all 10 tea leaves samples^52^. Their daily drinking water was also analyzed in Toyama Prefectural University, but no neonicotinoids were detected (Unpublished data). Therefore, rice, vegetables, fruits, or milk are suspected. Dinotefuran and thiacloprid which had not been registered in Sri Lanka in 2015 might be present as a contaminant in imported food or in domestic food through the use of illegally imported pesticides.

**Figure 2:**
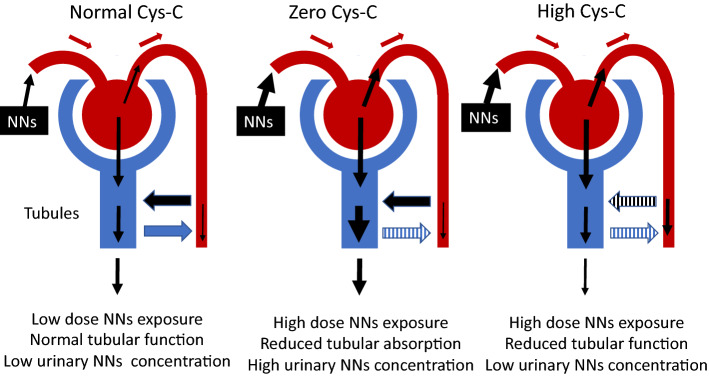
Candidate mechanism of lower urinary neonicotinoids (NNs) concentration in higher urinary Cystatin-C participants and in very low urinary Cystatin-C participants. This figure was originally made by Kumiko Taira with Power Point, following the software’s attribution guidelines.

The urinary detection of neonicotinoids was related to the renal tubular function represented by urinary Cystatin-C. In the High CysC group, a higher level of dinotefuran was detected, while a previous study had reported lower urinary neonicotinoid concentration in CKDu patients than in healthy volunteers living in non-CKDu affected area^26^. Neonicotinoids’ excretion in urine might decrease by the progression of CKDu, even if they were one of the risk factors (Fig. 2). To elucidate this pathophysiological question, hair and blood analysis to evaluate xenobiotic exposure in an epidemiological setting could be of considerable value. In the Zero CysC group, a rather high level of neonicotinoid detection and quantification were observed. Neonicotinoids and metabolites distributing around distal tubules directly by export arteriole or being reabsorbed by distal tubules with water, might cause an immunological reaction in the parenchyma and distal tubule dysfunction^53^. The symptoms in which they complained included uncommon symptoms of tubulointerstitial nephritis i.e. chest pains, recent memory loss, and auditory/visual hallucinations which might be caused by neonicotinoids.

Reportedly, a recent study showed self-harm intended thiamethoxam ingestion caused acute tubular damage after 2 days of being symptom free^54^. In addition, the pesticide formula contains some additives as surfactants and solvents, which are more toxic than the active substances^55–57^. Common neonicotinoid formulations contain renal toxic additives, such as dimethyl sulfoxide, *N*-methylpyrrolidone, diethylene glycol, propylene carbonate and mineral oil. Multiple acute kidney injury (AKI) episodes may cause CKD as the final stage of chronic renal pathological conditions^58^. Subacute and chronic neonicotinoids exposure may also cause tubular disorders. We previously reported in our experience that the consecutive intake of tea beverage and/or fruits contaminated with neonicotinoids may cause similar symptoms as acute intoxication (Table 3). In those cases, oliguria and the increase of urinary Cystatin-C were found^35^. Subacute or chronic occupational exposure of imidacloprid formulations caused renal disorders, such as hematuria and interstitial nephritis^59^. An animal study showed oral administration of imidacloprid at 0.6 mg/kg bw/day for 24 weeks in male mice caused tissue accumulation of imidacloprid and the metabolites in kidney as well as blood, testes, brain, lung, adipose tissues, liver, and pancreas^60^. Another animal study showed the oral administration of thiamethoxam at doses of 0.2 and 0.4 mg/kg/day for 15 days in male mice caused renal pathological changes in the parenchyma^53^. Additionally, CKD patients frequently complained of fever and neurological symptoms. They could be the nicotinic symptoms, but another possibility was that they were symptom of an infection, immunological disturbance, or chronic exposure to neurotoxic substances such as organophosphate insecticides and herbicides. Organophosphate insecticides, profenofos and diazinon seem to be the first line insecticides in Sri Lanka^48^. The herbicide glyphosate has secondary off-target toxicity in the mammalian brain and may cause limbic encephalopathy after occupational exposure^61,62^. In 2013, the Sri Lankan government banned four pesticides when renal toxicity had been reported, i.e. carbaryl, chlorpyrifos, carbofuran, and propanil^51^. They also banned glyphosate imported in October 2015 following a campaign over the fears the chemical causes CKD. However, after agricultural organizations pointed out there was no study linking CKD to glyphosate, so the import ban was lifted in July 2018; and its use was restricted to tea and rubber plantations^51^. We also found that acidic urine was prevalent in this area. It might be caused by high consumption of tea as a drink. Black tea leaves contain many organic acidic compounds, such as gallic acid, epigallocatechin gallate and other catechins^63^.

The limitations of this study are as follows: the small sample size, CKD diagnosis was not certified by a physician directly, the history of pesticides exposure in participants could not investigated thoroughly, no control area was set, no repetition of sampling was possible, and other neonicotinoids made in China were not investigated. Whether the seasonal change of DMAP detection in the urine was caused by the method of farming or food intake is unknown. We recommend that occupational and environmental exposure to neurotoxic pesticides through diet and application of pesticide formulations should be kept as low as possible in the CKDu-affected area and there should be greater surveillance of the routes of such exposure.

## Conclusion

We conducted a small-scale field-based descriptive study of urinary neonicotinoids/a metabolite, *N*-desmethyl-acetamiprid (the phase-I metabolite of acetamiprid) and symptoms in 15 CKD patients, 15 CKD patients’ families and 62 neighbors, in the Dry-zone of Sri Lanka in 2015. In the urine, *N*-desmethyl-acetamiprid (DMAP) was detected at the highest rate, followed by dinotefuran and thiamethoxam; and the detection levels in the CKD patients were lower than in the non-CKD participants. Urinary Cystatin-C elevation were frequently observed in male CKD participants, but extremely low levels or urinary Cystatin-C were observed in non-CKD participants with high urinary neonicotinoids quantification. CKD patients in the sampled areas exhibited more symptoms, and their complaints were more significant than the non-CKD participants, who appeared to have intoxication of neurotoxic xenobiotics including other type of pesticides. Urinary neonicotinoids may be one of the potential risk factors for renal tubular dysfunction in this area.

## Supplementary Information

Supplementary Information.
