# Supplementary material for: Urinary concentrations of neonicotinoid insecticides were related to renal tubular dysfunction and neuropsychological complaints in Dry-zone of Sri Lanka
Source: Sci Rep. 2021 Nov 18;11:22484. doi: 10.1038/s41598-021-01732-2 (PMC8602289; doi:10.1038/s41598-021-01732-2)
Supplement: Supplementary file 1 — Supplementary Information. [file 41598_2021_1732_MOESM1_ESM.pdf]

Urinary concentrations of neonicotinoid insecticides were related to renal tubular dysfunction and neuropsychological complaints in Dry-zone of Sri Lanka

TAIRA Kumiko, KAWAKAMI Tomonori, Sujithra Kaushaliya WERAGODA, H. M. Ayala S. HERATH, IKENAKA Yoshinori, FUJIOKA Kazutoshi, Madhubhani HEMACHANDRA, Nirmalie PALLEWATTA, AOYAMA Yoshiko, ISHIZUKA Mayumi, BONMATIN Jean-Marc, and KOMORI Makiko

Supplementary Tables S1. Candidate Etiology of CKDu in Sri Lanka.

Supplementary Table S2. Details of basic urinary findings and tubular biomarkers.

Supplementary Table S3. Pesticides sold by season in Mihintale, Anuradapura in 2015

Supplementary Table S4. Details of urinary neonicotinoids and *N*-desmethyl acetamiprid (DMAP) concentration in each group in May 2015 and in December 2015.

Supplementary Table S5. Comparison of detection rate more than LOQ and concentration of urinary neonicotinoids and DMAP (geometric mean) between three clinical categories.

Supplementary Table S6. Toxicological profile details of volunteers in High Cystatin-C group

Supplementary Table S7. Toxicological profile details of volunteers in Zero Cystatin-C group

Supplementary Table S8. Comparison of each urinary neonicotinoid concentration between the participants complained of each symptom and not complained of.

Supplementary Table S9. Comparison of the symptoms complained of between three clinical categories.

Supplementary Table S10. Comparison of the symptoms complained of between each class of urinary creatinine-adjusted Cystatin-C concentration

Supplementary Table S1. Candidate Etiology of CKDu in Sri Lanka.

Category	Details
Water contamination	metals/metaloids (fluoride, cadmium, arsenic, lead, uranium), hardness of water, mycotoxin, dissolved organic matter (DOM), interactions among ions (Ca^{2+}, PO_4^{-3}, F^-, and Mg^{2+})
Animal venoms	snake venoms
Chemicals	agrochemicals (insecticides, herbicides, and fertilizers)
Infection	Thailand orthohantavirus, leptospirosis
Lifestyle	dehydration, staple diet, antioxidant and micronutrient deficiencies, herbal medicine (aristolochia)
Genetic factor	

References

1. Rango, T., Jeuland, M., Manthrithilake, H. McCornick P. Nephrotoxic contaminants in drinking water and urine, and chronic kidney disease in rural Sri Lanka. *Sci. Total. Environ.* **518-519,** 574-85: 10.1016/j.scitotenv.2015.02.097 (2015).
2. Wasana, H.M., Perera, G.D., De Gunawardena, P.S., Bandara, J. The impact of aluminum, fluoride, and aluminum-fluoride complexes in drinking water on chronic kidney disease. *Environ. Sci. Pollut. Res. Int.* **22,** 11001-9; 10.1007/s11356-015-4324-y (2015).
3. Diyabalanage, S., et al. Has irrigated water from Mahaweli river contributed to the kidney disease of uncertain etiology in the dry zone of Sri Lanka? *Environ. Geochem. Health.* **38,** 679-90; 10.1007/s10653-015-9749-1 (2016).
4. Diyabalanage, S., Navarathna ,T., Abeysundara, H.T., Rajapakse S., Chandrajith, R. Trace elements in native and improved paddy rice from different climatic regions of Sri Lanka: implications for public health. *Springerplus.* **5,** 1864; 10.1186/s40064-016-3547-9 (2016).
5. Diyabalanage, S., Fonseka, S., Dasanayake, D.M.S.N.B., Chandrajith, R. Environmental exposures of trace elements assessed using keratinized matrices from patients with chronic kidney diseases of uncertain etiology (CKDu) in Sri Lanka. *J. Trace Elem. Med. Biol.* **39,** 62-70 (2017)
6. Mascarenhas, S., Mutnuri, S., Ganguly, A. Deleterious role of trace elements - silica and lead in the development of chronic kidney disease. *Chemosphere.* **177,** 239-249 (2017)
7. Dharma-Wardana, M.W.C. Chronic kidney disease of unknown etiology and the effect of multiple-ion interactions. *Environ. Geochem. Health.* **40,** 705-719; 10.1007/s10653-017-0017-4 (2018).
8. Nanayakkara S. *et al.* Systematic evaluation of exposure to trace elements and minerals in patients with chronic kidney disease of uncertain etiology (CKDu) in Sri Lanka. *J. Trace Elem. Med. Biol.* **54,** 206-213 (2019).
9. Ananda Jayalal, T.B., Jayaruwan Bandara, T.W.M.A., Mahawithanage, S.T.C., Wansapala, M.A.J., Galappaththi, S.P.L. A quantitative analysis of chronic exposure of selected heavy metals in a model diet in a CKD hotspot in Sri Lanka. *BMC Nephrol.* **20,** 208; 10.1186/s12882-019-1371-5 (2019).
10. Wasana H.M. *et al.* Drinking water quality and chronic kidney disease of unknown etiology (CKDu): synergic effects of fluoride, cadmium and hardness of water. *Environ. Geochem. Health.* **38,** 157-68; 10.1007/s10653-015-9699-7 (2016).
11. Wanigasuriya, K.P., Peiris, H., Ileperuma, N., Peiris-John, R.J., Wickremasinghe, R. Could ochratoxin A in food commodities be the cause of chronic kidney disease in Sri Lanka? *Trans. R. Soc. Trop. Med. Hyg.* **102,** 726-8 (2008).
12. Desalegn, B. et al. Mycotoxin detection in urine samples from patients with chronic kidney disease of uncertain etiology in Sri Lanka. *Bull. Environ. Contam. Toxicol.* **87,** 6-10 (2011).
13. Herath, H.M. et al. Chronic kidney disease in snake envenomed patients with acute kidney injury in Sri Lanka: a descriptive study. *Postgrad. Med. J.* **88,** 138-42; 10.1136/postgradmedj-2011-130225 (2012).
14. Herath,N. et al. Thrombotic microangiopathy and acute kidney injury in hump-nosed viper (Hypnale species) envenoming: a descriptive study in Sri Lanka. *Toxicon.* **60,** 61-5 (2012).
15. Silva, A., Samarasinghe, R., Pilapitiya, S., Dahanayake, N., Siribaddana, S. Viper bites complicate chronic agrochemical nephropathy in rural Sri Lanka. *J. Venom. Anim. Toxins. Incl. Trop. Dis.* **20,** 33; 10.1186/1678-9199-20-33 (2014).
16. Peiris-John, R.J., Wanigasuriya, J.K., Wickremasinghe ,A.R., Dissanayake, W.P., Hittarage A. Exposure to acetylcholinesterase-inhibiting pesticides and chronic renal failure. *Ceylon Med. J.* **51,** 42-3; 10.4038/cmj.v51i1.1382 (2006).
17. Jayasumana, C., Gunatilake, S., Senanayake, P. Glyphosate, hard water and nephrotoxic metals: are they the culprits behind the

epidemic of chronic kidney disease of unknown etiology in Sri Lanka? *Int. J. Environ. Res. Public Health.* **20,** 2125-47; 10.3390/ijerph110202125 (2014).

18. Jayasumana, C., Gajanayake, R., Siribaddana, S. Importance of Arsenic and pesticides in epidemic chronic kidney disease in Sri Lanka. *BMC Nephrol.* **15**,124 10.1186/1471-2369-15-124 (2014).
19. Jayasumana, C. *et al.* Drinking well water and occupational exposure to herbicides is associated with chronic kidney disease, in Padavi-Sripura, Sri Lanka. *Environ. Health.* **14,** 6 (2015).
20. Jayasumana, C. *et al.* Phosphate fertilizer is a main source of arsenic in areas affected with chronic kidney disease of unknown etiology in Sri Lanka. *Springerplus.* **4**, 90; 10.1186/s40064-015-0868-z (2015).
21. Jayasinghe, S., Lind, L., Salihovic, S., Larsson, A., Lind, P.M. High serum levels of p,p'-DDE are associated with an accelerated decline in GFR during 10 years follow-up. Sci. Total. Environ. **644,** 371-374; 10.1016/j.scitotenv.2018.07.020 (2018).
22. Makehelwala, M., Wei, Y., Weragoda, S.K., Weerasooriya, R., Zheng, L. Characterization of dissolved organic carbon in shallow groundwater of chronic kidney disease affected regions in Sri Lanka. Sci Total Environ. **660,** 865-875; 10.1016/j.scitotenv.2018.12.435 (2019).
23. Gamage, C.D., Sarathkumara, Y.D. Chronic kidney disease of uncertain etiology in Sri Lanka: Are leptospirosis and hantaviral infection likely causes? *Med. Hypotheses.* **91,**16-19 (2016)
24. Yoshimatsu, K. *et al.* Thailand orthohantavirus infection in patients with chronic kidney disease of unknown aetiology in Sri Lanka. *Arch Virol.* **164,** 267-271; 10.1007/s00705-018-4053-x (2019).
25. Herath, N.J. *et al.* Long term outcome of acute kidney injury due to leptospirosis? A longitudinal study in Sri Lanka. *BMC Res Notes.* **7,** 398; 10.1186/1756-0500-7-398 (2014).
26. Nanayakkara, I., Dissanayake, R.K., Nanayakkara, S. The presence of dehydration in paddy farmers in an area with chronic kidney disease of unknown aetiology. *Nephrology (Carlton).* **25,** 156-162; 10.1111/nep.13605 (2020).
27. Siriwardhana, E.A. *et al.* Dehydration and malaria augment the risk of developing chronic kidney disease in Sri Lanka. *Indian. J. Nephrol.* **25,** 146-51; 10.4103/0971-4065.140712 (2015).
28. Jayasekara, K.B. *et al.* Relevance of heat stress and dehydration to chronic kidney disease (CKDu) in Sri Lanka. *Prev. Med. Rep.* **15,** 100928; 10.1016/j.pmedr.2019.100928 (2019).
29. Wijesinghe, W., Pilapitiya, S., Hettiarchchi, P., Wijerathne, B., Siribaddana, S. Regulation of herbal medicine use based on speculation? A case from Sri Lanka. *J. Tradit. Complement. Med.* **7,** 269-271; 10.1016/j.jtcme.2016.06.009 (2016).
30. Siriwardhana, E.A. *et al.* Is the staple diet eaten in Medawachchiya, Sri Lanka, a predisposing factor in the development of chronic kidney disease of unknown etiology? - A comparison based on urinary β2-microglobulin measurements. *BMC Nephrol.* **15,** 103; 10.1186/1471-2369-15-103 (2014).
31. Nanayakkara, S. *et al.* Whole-exome sequencing reveals genetic variants associated with chronic kidney disease characterized by tubulointerstitial damages in north central region, Sri Lanka. *Environ. Health. Prev. Med.* **20,** 354-9; 10.1007/s12199-015-0475-1 (2015).
32. Kulathunga, M.R.D.L., Ayanka Wijayawardena, M.A., Naidu, R., Wijeratne ,A.W. Chronic kidney disease of unknown aetiology in Sri Lanka and the exposure to environmental chemicals: a review of literature. *Environ. Geochem. Health.* **41,** 2329-38; 10.1007/s10653-019-00264-z (2019).
33. Wimalawansa, S.J. Does fluoride cause the mysterious chronic kidney disease of multifactorial origin? *Environ. Geochem. Health.* 10.1007/s10653-019-00503-3 (2020).
34. Gunatilake, S., Sene, S., Orlando, L. Glyphosate's Synergistic Toxicity in Combination with Other Factors as a Cause of Chronic Kidney Disease of Unknown Origin. *Int. J. Environ. Res. Public Health* **16,** 2734; 10.3390/ijerph16152734 (2019).

Supplementary Table S2. Details of basic urinary findings and tubular biomarkers.

	May 2015			Dec 2015			
	CKDs	Families	Neighbors	CKDs	Families	Neighbors farmers	Neighbors not farmers
N	9	5	19	6	10	12	31
gravity ＜1.005	5 (55.6%)	2 (40%)	3 (15.8%)	No data	No data	No data	No data
pH, mean±SD	5.6±0.2	6.0±0.6	5.8±0.5	5.8±0.5	5.6±0.6	5.6±0.6	5.7±0.6
UACR >30	3 (33.3%)	0	1 (5.3%)	2 (33.3%)	0 in 9	0 in 11	0 in 29
Glucose >= (+)	0	0	0	0 in 5	0 in 9	1 in 11 (9.1%)	9 in 29 (31.0%)
Blood	3 (33.3%)	2 (40%)	3 (15.8%)	3 in 5 (60%)	7 in 9 (77.8%)	5 in 11 (45.5%)	20 in 29 (69.0%)
Creatinine <0.5g/L	4 (44%)	1 (20%)	3 (15.8%)	1 (18.7%)	5 (50%)	0	14 (45.0%)
Creatinine (g/L) mean±SD	0.72±0.57	0.92±0.58	1.21±0.65	1.12±0.87	0.65±0.37	1.20±0.63	0.87±0.77
Cystatin-C ＞70 μg/g Cre	2 (22.2%)	0	1 (5.3%)	3 (50%)	0	0	1 (3.2%)
Cystatin-C <0.01μg/L	0	1 (20%)	1 (5.3%)	0	0	0	5 (16.1%)
Cystatin-C mean±SD, median, IQR	270±734, 12.0, 11.0	3.4±2.7, 3.0, 7.0	18.4±45.8, 6.0, 204	1770±3400, 104, 3520	9.2±14.3, 4.2, 4.0	8.8±7.2, 5.9, 8.1	14.0±27.7, 5.8, 11.2
L-FABP > 8.4 μg/g Cre	3 in 8 (37.5%)	0 in 3	1 in 16 (6.3%)	2 in 5 (40%)	0 in 9	0 in 9	3 in 18 (16.6%)

Supplementary Table S3. Pesticides sold by season in Mihintale, Anuradapura in 2015

Period	Commercial name	Active ingredient	Type
April to May	Ceypetco Glyphosate36%SL	Glyphosate	Herbicide
June to July	Ceypetco M.C.P.A 40%SL	M.C.P.A	Herbicide
	Ceypetco M.C.P.A 60%SL	M.C.P.A	Herbicide
	3-4 DPA 36% E.C.	Propanil	Herbicide
	Gulliver	Azimsulfuron 50%	Herbicide
August	Avimavar Imduchloride	Imidacloprid	Insecticide
	Mospilan	Acetamiprid	Insecticide
	Marshal 20SC	Carbosulfan	Insecticide

Supplementary Table S4. Details of urinary neonicotinoids and *N*-desmethyl acetamiprid (DMAP) concentration in each group in May 2015 and in December 2015.

Studied period	Studied group	N	detected neonicotinoids	>LOQ (%)	Cre-adjusted concentration (μg/gCre)	
					Mean ± SD	Median, maximum
May	CKD patients	9	DMAP	55.6	0.48 ± 0.57	0.55, 1.78
			Dinotefuran	22.2	0.15 ± 0.30	<LOQ, 0.74
	Family members	5	DMAP	80	0.31 ± 0.28	0.23, 0.72
			Dinotefuran	20	0.02 ± 0.05	<LOQ, 0.11
	Neighbors	19	DMAP	89.5	0.56 ± 0.58	0.40, 1.88
			Dinotefuran	15.8	0.03 ± 0.10	<LOQ, 0.32
			Thiamethoxam	21.1	0.46 ± 1.64	<LOQ, 7.10
			Clothianidin	10.5	0.06 ± 0.19	<LOQ, 0.81
Dec	CKD patients	6	DMAP	100	1.39 ± 1.58	0.56, 3.93
			Dinotefuran	50	0.51 ± 0.90	0.03, 2.26
			Thiacloprid	16.7	0.04 ± 0.11	<LOQ, 0.26
			Imidacloprid	16.7	0.33 ± 0.80	<LOQ, 1.96
	Family members	10	DMAP	100	1.10 ± 0.77	1.12, 3.03
			Dinotefuran	20	0.16 ± 0.36	<LOQ, 1.06
			Thiamethoxam	20	0.32 ± 0.83	<LOQ, 2.62
			Clothianidin	10.0	0.02 ± 0.06	<LOQ, 0.18
			Thiacloprid	10.0	0.01 ± 0.03	<LOQ, 0.08
	Neighbors farmers	12	DMAP	91.7	0.67 ± 0.75	0.30, 2.03
			Dinotefuran	16.7	0.07 ± 0.16	<LOQ, 0.46
			Thiamethoxam	25.0	0.13 ± 0.27	<LOQ, 0.78
			Clothianidin	8.3	0.06 ± 0.20	<LOQ, 0.68
	Neighbors not farmers	31	DMAP	100	3.79 ± 5.62	1.39, 21.45
			Dinotefuran	12.9	0.50 ± 2.13	<LOQ, 11.51
			Thiamethoxam	22.6	0.34 ± 0.70	<LOQ, 2.51
			Clothianidin	16.1	0.18 ± 0.67	<LOQ, 3.61
			Thiacloprid	3.2	0.04 ± 0.21	<LOQ, 1.18
			Imidacloprid	3.2	0.03 ± 0.15	<LOQ, 0.83

Supplementary Table S5. Comparison of detection rate more than LOQ and concentration of urinary neonicotinoids and DMAP (geometric mean) between three clinical categories.

		CKDs (A)	Families (B)	Neighbors (C)	P value[a] (A vs BC)	P value[a] (B vs C)
		N=15	N=15	N=62		
Detection rate >LOQ%	DMAP	73.3%	93.3%	95.2%	**0.007**	0.77
	Dinotefuran	33.3%	20%	14.5%	0.11	0.56
	Thiamethoxam	0	13.3%	22.6%	0.05	0.43
	Clothianidin	0	6.7%	12.9%	0.16	0.50
	Thiacloprid	6.6%	6.7%	1.6%	0.42	0.27
	Imidacloprid	6.6%	0	1.6%	0.19	0.62
Concentration, uncorrected (μg/L)	DMAP	0.29	0.37	0.54	0.41	0.29
	Dinotefuran	0.13	0.09	0.08	**0.009**	0.94
	Thiamethoxam	<LOQ	0.09	0.10	0.32	0.52
	Clothianidin	<LOQ	0.07	0.08	0.19	0.28
	thiacloprid	0.03	0.03	0.03	0.29	0.079
	Imidacloprid	0.31	0.25	0.25	**0.031**	0.74
Concentration, Cre-adjusted (μg/gCre)	DMAP	0.48	0.43	0.69	0.29	0.23
	Dinotefuran	0.22	0.14	0.10	0.89	0.69
	Thiamethoxam	<LOQ	0.14	0.13	0.21	0.64
	Clothianidin	<LOQ	0.11	0.10	0.34	0.41
	thiacloprid	0.05	0.05	0.03	0.97	0.73
	Imidacloprid	0.61	0.59	0.79	0.36	1.00

[a] Chi-square test for categorical data, and t-test for numerical data

Supplementary Table S6. Toxicological profile details of volunteers in High Cystatin-C group

Case	age	sex	Clinical category	pH	blood	Cre (mg/dL)	Cys-C (μg/g Cre)	L-FABP (μg/gCre)	Neonicotinoid (μg/L)	Symptoms
1	65	M	Neighbor Not farmer	ND	+	20.2	152	37.4	DMAP 0.46	General fatigue, Palpitation, Recent memory loss, Anger, High urine volume
2	53	M	neighbor	5.5	-	153.9	204	18.1	DMAP 0.68	General fatigue, Recent memory loss, Altered consciousness, Low urine volume,
3	63	M	CKD	5.5	-	55.1	142	40.5	None	General Fatigue, Headache, Chest pains, Palpitation, Muscle symptoms, Cough, Fever, Recent memory loss, Altered consciousness, Dreamy state, Agitation, Auditory hallucination, Visual hallucination, Skin eruption, Sleepless, Nightmare, Restless, Edema, High urine volume, Appetite loss
4	58	F	CKD	5.5	+	72.9	193	35.6	DIN 1.65<br>DMAP 2.04	General fatigue, Headache, Palpitation, Stomachache, Muscle symptoms, Cough, Fever, Finger tremor, Dreamy state, Dizziness after stand up, Restless, Appetite loss, Reduce body weight
5	80	M	CKD	6.5	+	90.7	1849	ND	IMI 0.07<br>DIN 0.67<br>DMAP 0.48	General fatigue, Muscle symptoms, Cough, Recent memory loss, Altered consciousness, Skin itching, Constipation
6	48	M	CKD	6.0	+	32.5	2223	157	DMAP 0.18	No given answer correctly
7	61	M	CKD	ND	-	59.9	8561	107	DMAP 0.27	General fatigue, Muscle symptoms, Finger tremor, Recent memory loss, Anger, Auditory hallucinations, Restless, High urine volume, Appetite loss, Reduced body weight

ND: no data

DIN: dinotefuran, DMAP: n-desmethyl acetamiprid, IMI: imidacloprid

Supplementary Table S7. Toxicological profile details of volunteers in Zero Cystatin-C group

Case	age	sex	Clinical category	pH	blood	Cre (mg/dL)	L-FABP (μg/gCre)	Neonicotinoid (μg/L)	Typical Symptoms[a]	Other Symptoms
1	52	F	family	6.0	+	10.4	No data	<LOQ	Chest pains<br>Stomachache<br>Skin eruption<br>Skin itching	General fatigue, Muscle symptoms, Recent memory loss, Dizziness after standing up, Edema, high urine volume<br>Joint pain
2	8	M	Neighbor Not farmer	7.0	-	14.7	No data	<LOQ	Chest pains<br>Stomachache	Headache, cough, anger, Muscle symptoms, Recent memory loss
3	31	F	Neighbor Not farmer	5.0	+	30.9	3.4	CLO 0.26, DMAP 6.63	Chest pains<br>Stomachache<br>Skin eruption<br>Skin itching	General fatigue, Headache, Palpitation, Muscle symptoms<br>Dreamy state, Anger, Increase body weight
4	42	F	Neighbor Not farmer	5.5	+	15.8	8.3	DIN 0.52<br>DMAP 2.16	Chest pains<br>Stomachache<br>Skin eruption<br>Diarrhea	General fatigue, Palpitation, Cough, Fever, Recent memory loss, Altered consciousness, Anger, Restless, Edema<br>Auditory hallucination, Visual hallucination
5	28	F	Neighbor Not farmer	5.5	-	10.0	12.5 ↑	TMX 0.25<br>DIN 1.15<br>DMAP 1.69	Chest pains	Headache, Auditory hallucination, Visual hallucination
6	49	F	Neighbor Not farmer	5	+	26.3	No data	THI 0.31<br>DMAP 0.48	Chest pains<br>Stomachache<br>Diarrhea	Headache, Restless, Edema,<br>Muscle symptoms, Recent memory loss, Auditory hallucination, Visual hallucination, Dizziness after standing up
7	44	F	Neighbor Not farmer	5.5	+	22.6	4.1	CLO 0.82<br>TMX 0.31<br>DMAP 2.37	none	General fatigue, Muscle symptoms, Recent memory loss, Altered consciousness, Dreamy state, Auditory hallucination,<br>Sleepless, restless

Note: All participants lived in Anuradhapura, UACR was less than 30 (not albuminuria). Case 1 and 2 were collected in May 2015, and others in December 2015. Urine specific gravity of Case 1 and 2 was less than 1.005. Reference value of L-FABP is less than 8.4 μg/g Cre.

CLO: clothianidin, DMAP: n-desmethyl acetamiprid, TMX: thiamethoxam, THI: thiacloprid

[a] Typical symptoms were the symptoms significantly more complained of in Zero CysC groups than Normal CysC groups.

Supplementary Table S8. Comparison of each urinary neonicotinoid concentration between the participants complained of each symptom and not complained of (p value, t test).

	N	%	Imidacloprid	Thiacloprid	Clothianidin	Thiamethoxam	Dinotefuran	DMAP
Recent memory loss	61	67.0	0.575	0.798	0.651	0.482	0.401	0.065
Muscle symptoms	53	58.2	0.257	0.979	0.267	0.613	0.820	0.314
General fatigue	48	52.7	0.296	0.234	0.074	0.240	0.649	0.146
Chest pain/ Palpitation	48	52.7	0.353	0.934	0.439	0.386	0.182	0.432
Anger	47	51.6	0.400	0.089	0.788	0.499	0.071	0.699
Headache	45	49.5	0.373	0.739	0.021*	0.063	0.873	0.844
Restless	31	34.1	0.194	**0.021**	0.510	0.332	0.891	0.870
Auditory hallucinations	30	33.0	0.440	**0.018**	0.658	0.342	0.744	0.946
Dizziness after standing up	30	33.0	0.440	0.353	0.280	0.629	0.880	0.792
Altered consciousness	26	28.6	0.126	0.662	0.860	0.398	0.312	0.341
Visual hallucinations	24	26.4	0.510	0.534	0.754	0.685	0.521	0.810
Edema	22	24.2	0.534	0.469	0.097	0.287	0.770	0.282
Sleepless	21	23.1	0.731	0.829	0.846	0.695	0.409	0.515
Increase body weight	21	23.1	0.079	0.189	0.939	0.854	0.459	0.240
Stomachache	20	22.0	0.522	0.501	0.785	0.260	0.118	0.614
Agitation	19	20.9	0.069	0.829	0.182	0.296	0.204	0.380
Cough	18	19.8	0.791	0.417	0.886	0.184	0.266	0.301
High urine volume	18	19.8	0.069	0.469	0.270	0.241	0.176	0.874
Dreamy state	17	18.7	**0.042**	0.927	0.436	0.435	0.246	0.112
Appetite loss	16	17.6	**0.035**	0.450	0.227	0.495	0.059	0.118
Skin itching (urticaria)	15	16.5	0.649	0.468	0.390	0.062	0.094	0.996
Nightmare	14	15.4	0.653	0.918	0.350	0.533	0.270	0.306
Reduce body weight	14	15.4	0.625	0.468	0.193	0.326	0.640	0.154
Finger tremor	13	14.3	0.668	0.524	0.254	0.389	**0.002**	0.993
Skin eruption	13	14.3	0.653	0.505	0.155	**0.013**	0.631	0.977
Fever	11	12.1	0.683	0.545	0.278	0.413	0.002*	0.473
Recent memory loss with compulsive behaviors	11	12.1	0.699	0.091	0.304	0.438	0.790	0.768
Fear	7	7.7	0.751	0.637	0.441	0.786	0.327	0.553
Low urine volume	4	4.4	0.791	0.694	0.536	0.917	0.236	0.972
Diarrhea	4	4.4	**<0.0001**	**0.002**	0.531	0.636	0.582	0.860
Sudden change of senses of smell	3	3.3	0.839	0.763	0.590	0.684	0.532	0.220
Abnormal behavior	3	3.3	0.839	0.763	0.979	0.913	0.532	0.635
Constipation	3	3.3	**<0.0001**	0.763	0.590	0.684	0.249	0.634

* indicate negative correlation.

Supplementary Table S9. Comparison of the symptoms complained of between three clinical categories.

Clinical symptoms	Clinical category			P value[a]	
	CKDs (A)	Families (B)	Neighbors (C)	A vs BC	B vs C
N	15	15	61		
	%	%	%		
General fatigue	80.0	53.3	45.9	0.11	0.50
Headache	66.7	66.7	41.0	0.30	0.16
Chest pain/Palpitation	66.7	66.7	52.5	0.59	0.34
Stomachache	33.3	33.3	21.3	0.50	0.31
Muscle symptoms	80.0	86.7	49.2	0.29	**0.049**
Cough	26.7	20.0	18.0	0.51	0.72
Recent memory loss with food diary	40.0	66.7	63.9	0.50	0.58
Finger tremor	46.7	13.3	6.6	**<0.001**	0.36
Fever	46.7	6.7	4.9	**<0.001**	0.73
Altered consciousness	40.0	40.0	24.6	0.42	0.24
Dreamy state	26.7	33.3	13.1	0.43	0.070
Recent memory loss with compulsive behaviors	26.7	6.7	9.8	0.076	0.74
Agitation	40.0	33.3	14.8	0.10	0.10
Fear	20.0	6.7	4.9	0.06	0.73
Anger	53.3	60.0	49.2	0.92	0.42
Sudden change of senses of smell	6.7	6.7	1.6	0.43	0.27
Auditory hallucinations	46.7	46.7	26.2	0.31	0.14
Visual hallucinations	20.0	20.0	27.9	0.66	0.63
Abnormal behavior	13.3	6.7	0.0	**0.019**	**0.042**
Dizziness after standing up	46.7	46.7	24.6	0.26	0.11
Skin eruption	6.7	20.0	14.8	0.39	0.55
Skin itching (urticaria)	26.7	20.0	13.1	0.29	0.45
Sleeplessness	20.0	33.3	21.3	0.79	0.31
Nightmare	13.3	20.0	13.1	0.91	0.45
Restlessness	53.3	33.3	29.5	0.16	0.62
Edema	33.3	40.0	18.0	0.43	0.081
Low urine volume	6.7	0.0	6.6	0.83	0.35
High urine volume	66.7	6.7	16.4	**<0.001**	0.42
Constipation	13.3	0.0	1.6	**0.019**	0.64
Diarrhea	13.3	0.0	3.3	0.071	0.51
Appetite loss	60.0	20.0	6.6	**<0.001**	0.11
Reduced body weight	53.3	20.0	6.6	**<0.001**	0.11
Increased body weight	20.0	33.3	21.3	0.79	0.31

[a] Chi-square test

Supplementary Table S10. Comparison of the symptoms complained of between each class of urinary creatinine-adjusted Cystatin-C concentration

Group	Zero CysC (D)	Normal CysC (E)	High CysC (F)	p value[a] D vs E	p value[a] E vs F
N	7	78	7		
	%	%	%		
General fatigue	57.1	51.3	85.7%	0.77	0.08
Headache	57.1	50.0	28.6	0.72	0.27
Chest pains	85.7	28.2	14.3	**0.002**	0.43
Palpitation	28.6	47.4	42.9	0.34	0.82
Stomachache	71.4	23.1	14.3	**0.006**	0.59
Muscle symptoms	57.1	61.5	57.1	0.82	0.82
Cough	42.9	15.4	42.9	0.07	0.068
Fever（＞37 centigrade)	14.3	11.5	28.6	0.83	0.2
Finger tremor	0.0	15.4	28.6	0.40	0.37
Recent memory loss	71.4	67.9	85.7	0.85	0.33
Altered consciousness	28.6	26.9	42.9	0.93	0.37
Dreamy state	28.6	17.9	28.6	0.49	0.49
Recent memory loss with compulsive behaviors	28.6	9.0	28.6	0.11	0.11
Agitation	0.0	25.6	14.3	0.13	0.41
Fear	0.0	9.0	0.0	0.41	0.41
Anger	42.9	55.1	28.6	0.53	0.18
Sudden change of senses of smell	0.0	3.8	0.0	0.60	0.6
Auditory hallucinations	57.1	32.1	28.6	0.18	0.85
Visual hallucinations	42.9	25.6	14.3	0.33	0.5
Abnormal behavior	0.0	3.8	0.0	0.60	0.6
Dizziness after standing up	28.6	35.9	14.3	0.70	0.25
Skin eruption	42.9	11.5	14.3	**0.022**	0.83
Skin itching (Urticaria)	42.9	14.1	14.3	**0.049**	0.99
Sleepless	28.6	23.1	14.3	0.26	0.59
Nightmare	0.0	15.4	14.3	0.26	0.94
Restless	42.9	32.1	42.9	0.56	0.56
Edema	42.9	24.4	14.3	0.28	0.55
Low urine volume	14.3	25.6	28.6	0.50	0.86
High urine volume	28.6	41.0	57.1	0.52	0.41
Constipation	0.0	3.8	14.3	0.60	0.21
Diarrhea	28.6	2.6	0.0	**0.002**	0.67
Appetite loss	0.0	16.7	42.9	0.24	0.09
Reduce body weight	28.6	33.3	28.6	0.80	0.80
Increase body weight	28.6	42.3	0.0	0.48	0.98

[a] Chi-square test
